# Health Tract. No. 91

**Published:** 1865

**Authors:** 


HEALTH TRACT. NO. 91.

### BACK-BONE.

As light as one feather is, it will soon become completely flattened, if a thousand other feathers are piled upon it. But when a living substance is steadily compressed, it is destroyed, it is "absorbed," in medical language, and disappears. If a bandage is strapped around the stoutest arm, the parts under it will be reduced to skin and bone in a few days, if the bandage is gradually tightened, and that, too, without causing any special inconvenience. The back-bone, the *spinal column*, is composed of twenty-four alternate layers of hard bone, and a kind of gristle, which is soft, pliant, and compressible, like so much India-rubber, between each two bones. In the ordinary work of a day, the whole weight of the erect body pressing upon these elastic cushions, compresses them to the extent, that a good-sized man will be half an inch shorter at bedtime than he was on first rising in the morning. But if a person gets into the habit of leaning to one side, as some do, by carrying one shoulder higher than the other, or from want of energy, to sit and stand and move erectly, or from actual bodily debility — from either of these causes, the pressure on the elastic cushions, the India-rubber plates between the spinal bones, will not only tend to make the side of the cushion toward which there is the leaning thinner, by means of the greater weight, but also, by the law of pressure and absorption, thinner; that is, the whole cushion will be wedge-like, the thinner part of the wedge being on the side to which there is the leaning. If this leaning is kept up too long, the whole cushion will be absorbed, the bones themselves will begin to touch, and be absorbed also. This is *spinal disease*. If the cure is attempted before the bones touch, before all the elastic cushion has been removed by absorption, it may be effected; but when attention to the subject has been delayed until the bones meet, then a cure is hopeless. The principle of cure, in curable cases, is to relieve the pressure, by bending over on the other side, thus allowing the cushion to rebound by force of its inherent elasticity; and as this bending on the other side promotes absorption there, promotes the thinning process, while the opposite side gets thicker by its rebound, the equilibrium is soon restored, although the patient may not be quite so tall as before. The obvious practical inference is, that a perfect preventive of spinal deformity of this nature is an habitual erect position. But to make this an easy and practicable thing, an active life must be commenced; the person should be constantly on horseback or on foot, walking or working, for then an erect position may be maintained without weariness; but to endeavor to maintain it while at rest, in sitting, reading, writing, or sewing, is an unendurable weariness, or an impossibility. Walking with the head downward, or with a staff or cane, promotes a stooping position, and brings on an appearance of old age prematurely, not only by the effects upon the structure of the spinal column, but by throwing the weight of the body on the chest, thus compressing the lungs, diminishing their capability of receiving an adequate quantity of pure air, thus gradually purifying the blood less and less perfectly, until the whole mass of it becomes imperfect, impure, and diseased; then slight causes carry a man to the grave. An absolute preventive of all this is an habitual, persistent attention to the following rules:

1. Walk with the toes thrown outward.
2. Walk with the chin slightly above a horizontal line, as if looking at the top of a man's hat in front of you, or at the eaves or roof of a house.
3. Walk a good deal with your hands behind you.
4. Sit with the lower portion of the spine pressed against the chair-back

